# Recovery of pathogens with implementation of a weight-based algorithm for pediatric blood cultures: an observational intervention study

**DOI:** 10.1186/s12887-024-04930-9

**Published:** 2024-07-09

**Authors:** Nicolay Mortensen, Martin Skaranger Kristiansen, Odd Alexander Tellefsen, Unni Mette Stamnes Köpp

**Affiliations:** 1 Department of Child and Adolescent Medicine, Soerlandet Hospital, Kristiansand, Norway; 2 Department of Microbiology, Haukeland University Hospital, Bergen, Norway; 3 Department of Microbiology, Soerlandet Hospital, Kristiansand, Norway

**Keywords:** Blood culture, Volume, Positivity rate, Contamination rate, Pediatric, Children

## Abstract

**Background:**

Recovering pathogenic bacteria and yeast from pediatric blood cultures and reliably distinguishing between pathogens and contaminants are likely to be improved by increasing the volume of blood submitted to microbiology laboratories for culturing beyond the low volumes that have historically have been used. The primary aim of this study was to assess whether the pathogen recovery rate would increase after implementation of a weight-based algorithm for determining the intended volume of blood submitted for culturing.

Secondary aims were to: 1) evaluate the effects of the algorithm implementation on the blood culture contamination rate; 2) determine whether pathogens might be found more often than contaminants in several as opposed to single bottles when more than one bottle is submitted; and 3) describe the microbiological findings for pathogens and contaminants in blood cultures by applying a clinical validation of true blood culture positivity.

**Methods:**

A pre-post comparison of positivity and contamination rates after increasing the theoretical blood volume and number of blood culture bottles was performed, on the basis of a clinical validation of blood culture findings as pathogens vs contaminants.

**Results:**

We examined 5327 blood cultures, including 186 with growth (123 true positives and 63 contaminated). The rate of true positive blood cultures significantly increased from 1.6% (42/2553) pre to 2.9% (81/2774, *p* = .002) post intervention. The rate of contaminated blood cultures did not change significantly during the study period (1.4% [35/2553] pre vs 1.0% [28/2774], *p* = .222) post intervention), but the proportion of contaminated cultures among all positive cultures decreased from 45% (35/77) pre to 26% (28/109, *p* = .005) post intervention. A microorganism that grew in a single bottle was considered a contaminant in 35% (8/23) of cases, whereas a microorganism that grew in at least two bottles was considered a contaminant in 2% (1/49, *p* < .001) of cases. According to common classification criteria relying primarily on the identity of the microorganism, 14% (17/123) of the recovered pathogens would otherwise have been classified as contaminants.

**Conclusion:**

Implementation of a weight-based algorithm to determine the volume and number of blood cultures in pediatric patients is associated with an increase in the pathogen recovery rate.

**Supplementary Information:**

The online version contains supplementary material available at 10.1186/s12887-024-04930-9.

## Background

Blood culture (BC) remains the core diagnostic tool to uncover the microbiological etiology of sepsis with bacteremia and fungemia. A positive culture enables physicians to provide a focused diagnostic work-up to identify the source of infection, pursue source control and tailor antibiotic treatment. Changes in epidemiology of BC findings in the era of vaccines and medical advances [1, 2] has prompted questions regarding the historical assumption that children require small volumes of blood for culturing, because of the high concentrations of microorganisms present in the bloodstream during septicemia [3, 4]. Low-level bacteremia is increasingly being recognized in the pediatric population [5, 6], and several studies have achieved a higher pathogen recovery rate from BCs by increasing the amount of blood incubated [7–9].

Several algorithms for determining the appropriate volume of blood are available, depending on the age and/or the weight of the child, as reviewed by Huber et al. [10]. Despite an emerging consensus regarding the need for optimizing BC collection in pediatric patients, the optimal volume of blood remains debated [10–12]. In addition to increasing the volume of blood, drawing more than one BC bottle could also inform clinicians and microbiologists of whether an isolated microorganism represents contamination or true bacteremia, particularly for immunocompromised individuals, or patients with indwelling central venous catheters [9].

BCs contaminated with non-pathogenic microorganisms can lead to unnecessary antibiotic treatment and prolonged hospital stays [13]. Several factors may decrease BC contamination, such as hygienic measures, educational programs and training of dedicated phlebotomist teams [14, 15]. Moreover, increasing blood volume could in itself decrease the contamination rate [7, 10, 16], probably because of the concentrations of contaminant skin bacteria relative to those of bacteria present in the bloodstream, when a sufficient volume is inoculated in BC bottles.

Most studies on BC positivity and contamination rates have used a schematic definition of pathogens and contaminants based solely on the isolated microorganism, without considering the clinical setting for each patient from which the microorganism was recovered [9, 17].

At the Department of Child and Adolescent Medicine at Soerlandet Hospital, a local hospital in the south of Norway, we aimed to increase pathogen recovery and decrease microbial contamination in pediatric BCs by implementing an algorithm that sought to increase the volume and number of BCs incubated according to the weight of the patient. We retrospectively evaluated this implementation by assessing the positivity and contamination rates before and after, and defined each finding as pathogen or contaminant according to a clinical validation, thus providing a true numerator in the calculation of positivity and contamination rates.

## Methods

### Study setting and design

The Department of Child and Adolescent Medicine at Soerlandet Hospital is located in the neighboring cities of Arendal and Kristiansand in southern Norway. The hospital serves as the local hospital for the county of Agder and has an uptake area of almost 70,000 children and adolescents between 0 and 18 years of age.

Our hospital used the Bact/ALERT 3D BC system from bioMérieux, which was replaced by the Bactec FX system from Becton Dickinson Microbiology Systems in May 2018 through public procurement, as part of a scheduled system replacement. The blood volumes in pediatric and regular bottles were identical between systems.

Before implementation, our hospital followed the practice of drawing a single 4 ml pediatric BC bottle for all children with suspected bloodstream infection before initiating antimicrobial treatment. This procedure was performed regardless of the weight and age of the child, and excluded, and continues to exclude, patients admitted to the Neonatal Intensive Care Unit from the delivery and maternity wards. In May 2017 a weight- and age-based algorithm (Table 1) for determining the volume of blood and number of cultures was implemented, by using the procedure suggested by the American Society for Microbiology and Infectious Disease Society of America [18] as a reference. The weight intervals were rounded to whole numbers to allow for sampling according to the age- and sex-based weight estimation algorithms commonly used in the emergency department. The study was performed as a pre-post intervention quality improvement study.

**Table 1:** Algorithm for volume of blood, number and type of BC bottles for pediatric patients at Soerlandet Hospital, Norway

Category	Culture set 1	Culture set 2	Total volume
Neonate and < 3 kg	1–3 ml	-	1–3 ml
3–12.9 kg	4 ml	2 ml	6 ml
13 kg to 12 years	10 ml aerobic	10 ml aerobic	20 ml
> 12 years	10 ml aerobic 10 ml anaerobic	10 ml aerobic 10 ml anaerobic	40 ml

### Aims

The primary aim of the study was to assess whether implementation of a weight-based algorithm for determining the volume of blood submitted for culturing might increase pathogen recovery. Secondary aims were to: 1) evaluate the effects of the algorithm on BC contamination rate; 2) determine whether pathogens grew more often than contaminants in several bottles when more than one bottle was submitted; and 3) describe the microbiological findings of pathogens and contaminants in BCs by applying clinical criteria for BC positivity.

### Inclusion and data collection

A BC was defined as the total amount of blood cultured at a specific point in time, and could consist of several sets of several bottles as shown in Table 1. Positive BCs were retrospectively identified through the hospital laboratory database from 2014 through April 2017 in the pre period, and from May 2017 through 2020 in the post period. Patients with any growth in a BC during the study period were included. A team consisting of a pediatric infectious disease physician (MK) and a medical microbiologist (OAT) reviewed the histories of patients included through electronic medical records. We extracted data on age, weight, sex and immune status. A patient was defined as immunocompromised if any of the following were present: current oncological chemotherapy, congenital or acquired immunodeficiency, immunosuppressant drugs, premature infants below 32 weeks’ gestational age in the first year of life and presence of a central venous catheter.

### Positivity and contamination rates

BCs with growth were defined as positive. A BC was defined as a true positive, regardless of the number of bottles with growth, if the patient had received targeted antimicrobial treatment for the identified microorganism, and both the history and biochemical or radiological tests were compatible with infectious disease, as judged by the review team. Only one positive BC per 30-days was included for each patient. Other cultures with growth were defined as contaminated. Positivity rates were calculated for all cultures with growth and for true positives, with the total number of BCs drawn in the corresponding time period as the denominator. Contamination rates were calculated for contaminated cultures among all cultures and among all positive cultures.

### Microbiology

Every identified microorganism was classified according to the highest taxonomic rank available up to the species level and grouped as shown in Supplementary Table 1. In the event of identification of several isolates in a single BC, only one microorganism per BC was included for reporting purposes. The complete set of findings is given in Supplementary Table 1.

### Statistics

Pearson’s chi-squared test was used for comparisons of categorical variables. Mann Whitney U-test was used for comparisons of continuous variables. Confidence intervals were set at 95%. Statistical analysis was performed in IBM SPSS Statistics, version 26, and MedCalc (www.medcalc.com, Ostend, Belgium).

## Results

Table 2. summarizes the numbers of BCs pre and post intervention, including the numbers of cultures with growth and the corresponding patient characteristics. The rate of true positives divided by the total number of cultures drawn significantly increased after implementation of the weight-based algorithm. The overall positivity rate also increased, although not significantly. Although the contamination rate, calculated with the total number of cultures drawn in the denominator, appeared to be relatively unchanged post intervention, the proportion of contaminated cultures among all positives in the post period decreased from 45% (35/77) to 26% (28/109, *p* = 0.005).

**Table 2.** Positivity and contamination rates of BCs, including patient characteristics, before and after implementation of a weight-based algorithm for determining the volume and number of cultures at Soerlandet Hospital, Norway

	**Total**		**Pre**		**Post**		***p***-value
All cultures, N (%)	5327	(100)	2553	(100)	2774	(100)	-
No growth, n (%)	5141	(96.5)	2476	(97.0)	2665	(96)	-
Growth, n (%)	186	(3.5)	77	(3.0)	109	(3.9)	.070
Positive, n (%)	123	(2.3)	42	(1.6)	81	(2.9)	**.002**
Contamination, n (%)	63	(1.2)	35	(1.4)	28	(1.0)	.222
Age, years, median (IQR)	2.0	(0–9)	2.0	(0–10)	2.0	(0–9)	.690
Age groups^a^, n (%)							
Newborn, 0 - 28d	26	(14)	8	(10)	18	(17)	.301
Infant, 29d - 11m	56	(30)	25	(32)	31	(28)	.667
Toddler, 12 - 35m	30	(16)	12	(16)	18	(17)	.886
Preschool, 3 - 5y	18	(10)	6	(8)	12	(11)	.507
School, 6 - 12y	29	(16)	14	(18)	15	(14)	.487
Teenager, >13y	27	(15)	12	(16)	15	(14)	.222
Female, n (%)	70	(40)	24	(32)	49	(44)	.058
Weight, kg, median (IQR)	13.0^b^	(6.6–31.0)	13.0	(5.9–33.0)	13.1	(6.8–28.3)	.652
Immunocompromised, n (%)	19	(11)	6		16		.154

^a^Time units: Days (d), months (m), years (y)

^b^Two missing cases pre intervention; three missing cases post intervention

Among all BCs with growth, more than one bottle was drawn in 16% (12/77) of cases before implementation, as compared with 55% (60/109, *p* ≤ 0.001) after implementation. In the 39% (72/186) cases in which at least two bottles were drawn across the study period, an identified microorganism that grew in only one bottle was considered a contaminant in 35% (8/23) of cases, as compared with 2% (1/49, *p* < 0.001) of cases when an identified microorganism grew in at least two bottles.

**Fig. 1:**
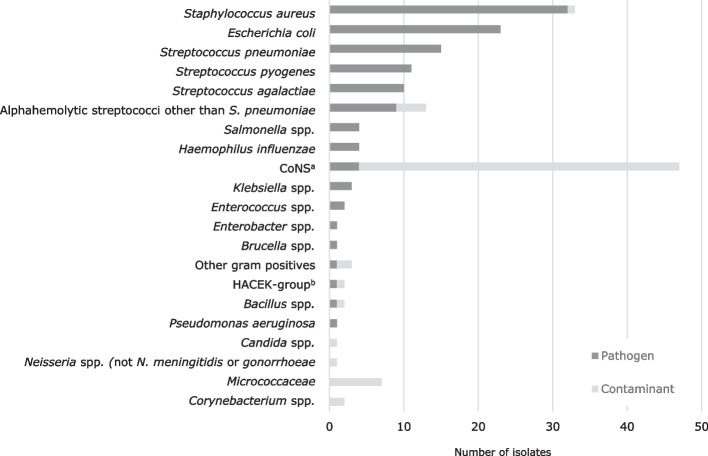
Pathogens and contaminants identified from *n* = 186 pediatric BCs between 2014 and 2020 at Soerlandet Hospital, Norway. ^a)^ Coagulase-negative staphylococci. ^b)^*Haemophilus, Aggregatibacter, Cardiobacterium, Eikenella, Kingella*

Figure 1 shows the number of identified microorganisms sorted by group, according to Supplementary Table 1, and labeled as pathogen vs contaminant. One case of *Candida* spp. was classified as a contaminant, because it was isolated from a patient with eczema herpeticum, no clinical or biochemical suspicion of septicemia and no indwelling catheters. A similar situation was observed in a case of *S. aureus* that was identified together with three coagulase negative staphylococci (CoNS) in an infant with dehydration fever. The results from all BCs with growth are shown in Supplementary Table 1, including every identified microorganism for which two or more species were isolated from a single sample.

## Discussion

We conducted a quality improvement study assessing whether the pathogen detection rate would improve in pediatric blood cultures after implementation of an algorithm that sought to increase the volume and number of BCs drawn according to the patient’s weight. Our findings indicated that the rate of true positives significantly increased, and the proportion of contaminated cultures among all positives decreased, with implementation of the algorithm. A recovered microorganism was more often considered a contaminant if it was present in only a single bottle among a multi-bottle set of cultures. Additionally, our findings highlight how microorganisms commonly dichotomized as pathogens (e.g., *S. aureus* and yeast) or contaminants (e.g., CoNS) solely according to their identity, can act as pathogens or contaminants depending on the clinical setting.

Our study supports findings in published reviews [10–12] and studies [7–9] calling for increasing the volume of blood submitted for pediatric BCs beyond that historically used, to achieve a higher pathogen recovery rate. However, even though the theoretical volume of blood drawn from a patient is increased by the algorithm as compared to the governing practice at our institution prior to implementation, and the number of multi-bottle sets significantly increased across the evaluation period, the actual volume of blood drawn was not measured and serves as a major limitation. Further studies are required to assess the appropriate volume required to achieve an optimal pathogen recovery from BCs without compromising patient safety.

Achieving an adequate volume of sample material is inherently challenging in pediatric BCs because of several factors but is of utmost importance, because an adequate blood volume, in addition to increasing pathogen recovery, could decrease contamination, as previously reported [7, 10]. Because specimen collection practices at our institution were not altered during the study period, the decrease in the proportion of contaminated BCs was likely to have been due to the increase in volume. However, we cannot rule out potential effects of awareness of technical factors or patient selection contributing to contamination among our colleagues who performed sample collection.

The use of clinical validation of a positive versus contaminated culture is a strength of our study. In a few cases, the classification of pathogen versus contaminant was made subjectively by the review team, which can make the results more challenging to compare with others. Although most microorganisms commonly found in BCs are often considered pathogens or contaminants according to taxonomic identity alone [19], a categorical approach to microorganism classification might fail to elucidate the duality of certain microorganisms that are considered either contaminants or pathogens according to clinical circumstances. By extension, applying a generic classification would also affect the numerator in determining both positivity and contamination rates. Among all positive cultures in our study, 14% (17/123) of pathogens would have been classified as contaminants according to common classification criteria, as discussed by Hall et al. [20] and studies based on surveillance data [21]. These cultures notably included four cases of CoNS, an emerging and heterogeneous group of bacteria increasingly responsible for nosocomial infections [22, 23].

Our study has several limitations. Even though the theoretical volume of blood drawn from a patient is increased by the algorithm as compared to the governing practice prior to implementation, the actual volume of blood drawn was not measured as previously discussed. Both BC systems used by our lab during the study period uses bottles that by vacuum automatically should fill with the appropriate volume of blood, aided by visual assessment by the health care worker that performs the sampling to avoid over-filling. Additionally, our lab has internal routines in place to ensure that BC bottles are appropriately filled before being incubated. Despite these factors and others, it is still likely that optimal volumes are not reached in several cases as reported by Connell [24], and this is highlighted in our material as well by the number of patients with a single BC bottle drawn despite what is warranted by the algorithm, as discussed in the above.

Although the median age of patients with positive cultures did not differ significantly in our study population, age still might have been a confounder for both positivity and contamination rates. Increasing the number of dedicated venipunctures required to obtain cultures, as defined by the algorithm, could raise the sampling threshold, particularly among small children, in whom BC collection might be particularly painful or technically challenging. Because small children have been reported to have the lowest positivity rates combined with higher contamination rates [25, 26], use of this algorithm might have selected patients with a higher pretest likelihood of bacteremia and thereby skewed the results. Because the time intervals for pre and post intervention were not balanced, the total number of BCs appeared to decrease post intervention, thus implying a narrowing of the indication for BC sampling by the attending health care professionals. We do not account for false negative BCs. With the sensitivity of BCs to detect bacteremia being dependent on the volume of blood incubated, the prevalence of false negative BCs could be larger in the pretest group. Also, the review team was not blinded to whether a sample was drawn pre or post intervention, which could bias the clinical validation.

The scheduled replacement for BC incubator system occurred within the study period and might have affected the results. Studies on adult patients comparing Bact/ALERT 3D from bioMérieux and Bactec FX from Becton Dickinson Microbiology Systems have reported variations in test result parameters but have nonetheless concluded that the systems have comparable performance [27–30].

## Conclusion

We conclude that implementing a weight-based algorithm to determine the intended volume and number of BCs for pediatric patients beyond the low volumes that historically has been common practice, yields a higher pathogen recovery2. A clinical approach to classification of microorganisms present in BCs may aid in clarifying equivocal findings. Further studies are needed to establish the optimal volume of blood, while also considering patient safety and the technical feasibility of sample collection for health care professionals.

### Supplementary Information

Supplementary Material 1.

## Data Availability

Data supporting the findings are provided in the article and its supplementary materials. Because of the small sample size and privacy concerns, data on individual participants´ sex, age and immune status are not reported or made available.
